# Dating App Safety Among Older Women in Australia: Using Informational Videos for Effective Safety Messaging Delivery

**DOI:** 10.1177/10778012251366224

**Published:** 2025-08-07

**Authors:** Emma L. Turley, Jan Cattoni, Nichola Corbett-Jarvis

**Affiliations:** 1 College of Psychology, School of Health, Medical, and Applied Sciences, 6939 Central Queensland University, Brisbane, Queensland, Australia; 2 School of Education and the Arts, 6939 Central Queensland University, Cairns, Queensland, Australia; 3 6939 College of Law, School of Business and Law, 6939 Central Queensland University, Cairns, Queensland, Australia

**Keywords:** dating apps, older women, education, intervention

## Abstract

This study investigates dating app safety concerns and effective safety messaging for Australian women aged 55 and over. As older women increasingly use dating apps for companionship, romance, or casual encounters, they may face risks such as harassment and scams. Using a qualitative approach, 121 cisgender women viewed three character-driven educational safety videos and then completed a survey. Reflexive Thematic Analysis generated four themes: safety concerns, modalities of safety messaging, video-based knowledge building, and behavioral change. Findings can be used to inform effective intervention development for targeted groups of people, providing memorable visual alternatives to traditional text-based safety information.

## Introduction

Over the last decade and a half, the proliferation of dating apps has transformed the landscape of contemporary relationships, transforming how people meet, connect, and form romantic or casual partnerships (Portolan & McAlister, 2022). Dating apps offer unprecedented accessibility and convenience, allowing individuals to browse potential matches and communicate and arrange dates with matches via their smartphones (De Ridder, 2022). As a result, these digital platforms have witnessed a staggering rise in popularity globally, catering to a diverse range of demographics and relationship preferences, from casual hookups to long-term relationships and marriages (Portolan & McAlister, 2022).

Despite the fact that older people aged 60+ are the fastest growing group of online dating users (Ellin, 2014), there is limited research that explores their perceptions about, and experiences of, dating apps, risk, and safety. This article will document a study aimed at exploring the dating app safety concerns and modalities of effective safety messaging for Australian women aged 55 years and over. We will begin with an overview of dating app use in mid and later life, before discussing the risks of harassment and violence facilitated by dating apps and the implications of this for women in this demographic in Australia. From this point, we will use the term “older women” to mean women 55 years and older.

Dating apps replicate, and often amplify, the normative expectation that women engage in “safety work” to protect themselves from harm, which encourages women to seek information about how they can recognize, minimize, and respond to risk when using dating apps (Berkowitz et al., 2021; Gillet, 2023; Vares, 2024). For older women who are new to or are considering using dating apps, such educational resources may fail to provide guidance in a user-friendly manner because the modality and content are unsuitable for the cohort. In response to this apparent gap in resources, academics in arts, law, and criminology collaborated with external partners to produce a series of educational videos tailored to the needs of this particular audience, which included character-driven storylines and humor. Participants were asked to watch the three educational videos and respond to a series of qualitative questions. This aspect of the study therefore aimed to identify how educational resources can be tailored to meet the unique needs of this cohort in addition to providing insight into older women's experiences and perceptions concerning their use of dating apps, which is currently unexplored in the literature.

### Dating Apps for Older People

The use of dating apps to meet sexual and/or romantic partners has become ubiquitous around the world, and the stigma once attached to online dating has begun to fade (Sharabi, 2023). In fact, online dating, including the use of dating apps, has become the most common method to meet romantic and sexual partners (Albury et al., 2020). Dating apps allow access to a range of relationship typologies, including long- and short-term romantic partnerships, casual dates, and one-time sexual hookups (Portolan & McAlister, 2022). Australian singles commonly use dating apps to facilitate these relationships (Bailey, 2012). Rates of dating app use in Australia have increased over the last decade and it is estimated that 52% of Australians have used a dating app (Tan, 2017). The shift to app-based technology featuring geospatial location capabilities has made online dating more accessible than ever for users (Portolan & McAlister, 2022), and there are around 4 million Australians registered with at least one dating app (Relationships Australia, 2017).

Research focusing on the use of dating apps usually focuses on a younger demographic of adults (e.g., Byron et al., 2021; Debnam & Kumodzi, 2021; Hammack et al., 2022; Mignault et al., 2022; Pym et al., 2021). However, the dynamics of later-life romantic relationships are likely to differ from younger people's relationship dynamics (Dickson et al., 2005). There is limited research that examines online dating for mid- and later-life adults, including those who have experienced a relationship breakdown or widowhood (Dwyer et al., 2021; McWilliams & Barrett, 2014). The majority of research exploring relationships in this age group focuses on sexual function, medical issues, and research within long-term marriages, meaning there is a lack of focus on single, older adults who are likely to have different dating experiences (Malta & Farquharson, 2014). Around one and a half million single women in Australia are aged 55 and over (ABS, 2022), which, coupled with an ageing population reporting high levels of loneliness, suggests that there will be an increasing number of older women using dating apps to facilitate relationships (AIHW, 2023; Franklin et al., 2019). Data demonstrates that dating apps are currently most popular among the 35–54 year cohort (Smith & Anderson, 2016), therefore it is likely that this group will continue to use dating apps as they age, thus further increasing the use of dating apps among women aged 55 or older over time.

### The Appeal of Dating Apps

Older people are more frequently using technology to enhance social interactions and engagement and to reduce loneliness (Francis et al., 2019; Marston, 2019), but as we highlighted earlier, there is a lack of research on dating apps use among older demographics (Marston et al., 2020). This is likely due to the negative perception around sex and intimacy for older adults and the stereotyped notion that this demographic loses interest in romance, intimacy, and sex in later life (Calasanti, 2008; McWilliams & Barrett, 2014; Wada et al., 2015). Despite this perception, many older adults are looking for, and engaging in, new later-life sexual and romantic relationships (Malta & Farquharson, 2014), and are therefore using dating apps to bolster sparse dating markets (Dwyer et al., 2021). There are myriad reasons that older people's “dating pools” have become thin, including lack of access to potential partners, limited social lives, loss of friendship networks, and long working hours or delayed retirement (Dwyer et al., 2021). In addition, older people report feeling disillusioned with offline meeting spaces, such as bars, and feel unwelcome and out of place when they attend (Dwyer et al., 2021; McWilliams & Barrett, 2014). Women specifically have fewer dating opportunities as single women outnumber single men, and men are more likely to be married (Kinsella & He, 2009).

Research suggests that online dating extends the opportunities to date for older adults along with broadening the pool of potential partners beyond real-life social spheres (Malta & Robards, 2017; Vandeweerd et al., 2016). In addition, dating apps can circumvent difficulties in meeting face to face and via offline social networks (Rosenfeld & Thomas, 2012). For women in this demographic, dating apps provide a means to break down communication barriers and redefine social norms and rules around dating convention (Malta & Robards, 2017; Malta, 2013), which was particularly advantageous for women who were socialized into a more passive and gendered dating role in their younger life (McWilliams & Barrett, 2014). Online dating appeals to older women as it provides them more control over the dating process, including opportunities to try out relationships before meeting in person, move at their own pace, and exit or terminate relationships more easily (Malta & Farquharson, 2014; Malta & Robards, 2017; Marston et al., 2020; McWilliams & Barrett, 2014), which reduces feelings of vulnerability when compared with initiating or maintaining contact in person (Ben-Ze’ev, 2004). However, the dating app landscape is not a dating utopia for women. Alongside the benefits outlined in this section, apps can facilitate sexual harms (Byron & Albury, 2018). In the next section, we discuss the sexual harassment and violence that can be perpetrated against women via dating app platforms.

### Dating App-Facilitated Violence

Harm, abuse, and violence that is perpetrated via digital communications technology is described in a variety of ways in the literature, including technology-facilitated sexual violence and abuse (TFSVA), dating app-facilitated sexual violence and abuse (DAFSVA) and cybervictimization (see Henry et al., 2020 and Powell & Henry, 2016 for a comprehensive overview of terminology). Following Stanko (1990) and Kelly (1987), we acknowledge the importance of examining sexual harm on a continuum rather than through a binary lens, and as such we recognize the diversity of harms that can be caused to dating app users.

TFSVA is pervasive on dating apps and websites. A survey of 9,987 dating app or website users found that 72.3% of respondents experienced at least one form of DAFSVA within the last 5 years (Wolbers et al., 2022). DAFSVA ranged from abusive and threatening language, harassment, stalking, and receipt of unsolicited images to in-person violence and the taking of images/recordings without consent once they met the other user offline (Wolbers et al., 2022). Notably, TFSVA is gendered in nature (Cama, 2021). Men are more likely to perpetrate sexual harms while women report significant distress as victim-survivors of TFSVA (Powell & Henry, 2019). Gillet's (2019) work examined women's experiences of everyday violence on the dating app Tinder, highlighting frequent unsolicited sexual images, such as “dick pics,” unwanted requests for sex, abuse based on gender and sexuality, and sexual assault. Harassment and violence against women facilitated via dating apps is not rare, with 1 in 10 Australian women reporting an unwanted sexual experience with someone they first met online (Powell & Henry, 2017), due to an unclear and ambiguous process of consent (Furlo et al., 2021; Turley et al., 2023). Women also described how, due to the unique context of dating app meetings, it was difficult to refuse sex after meeting via an app (Lauckner et al., 2019), demonstrating that sexual harms in the offline world are facilitated through app technology (Rowse et al., 2020).

Sexual harassment and violence frequently move from the online to the offline, real-world context at subsequent face-to-face meetings after connecting via a dating app (Gewirtz-Meydan et al., 2024). In fact, some perpetrators deliberately use dating apps as a method to procure victims to sexually assault, rape by proxy, blackmail, and coerce (Henry et al., 2020). The U.K. National Crime Agency (2016) states that the number of people who report being raped on a first face-to-face date after meeting via a dating app increased sixfold in 5 years. The geolocation capacity of dating apps allows predators to locate women to abuse, adding an additional element of risk even when no face-to-face meetings are planned (Henry & Powell, 2018). Abuse is not limited to sexual harm. There is also the risk of catfishing, blackmail, and financial and romance scams perpetrated via dating apps (Gewirtz-Meydan et al., 2024).

Well before the technological evolution of dating, “rape culture” existed to reinforce the responsibility of women to protect themselves from rape (Powell & Henry, 2017). Societal norms and attitudes that trivialize and normalize violence against women and minimize the responsibility of men in such cases are replicated in the online dating environment, where patriarchal heterosexual frameworks generally steer behavior (Gillet, 2021; Licoppe, 2019; Lindsay, 2015). However, as discussed above, the unique functions of dating apps may intensify the user's experience of harm (Henry et al., 2020). In addition, media narratives often highlight the use of dating apps as inherently risky (Albury et al., 2020). Often, publications conflate social and public health concerns, abusive and violent behavior, scams, mental and sexual health issues, discrimination, and racism with dating apps (Albury et al., 2020). The prevalence of this narrative appears to foster a culture of fear in women using dating apps, resulting in women often engaging in “safety work” in an attempt to reduce the risk of harm they perceive may occur both online and when meeting potential matches offline (Berkowitz et al., 2021; Farvid & Aisher, 2016; Gillet, 2023, 2021; Vares, 2024). Such concerns are amplified due to limited support from dating app platforms around maintaining safe connections, privacy, reporting negative on-app and off-experiences with other app users, scams, and fake profiles. Although it should never have to be the responsibility of women to protect themselves from violence and harm, engaging in “safety work” has become normalized in the dating app environment to mitigate perceived risks associated with utilizing such apps (Vares, 2024).

### The Current Study

There is limited research examining safety and risk as applied to older age cohorts of people using dating apps. Malta and Robards (2017) suggest that older people may not be aware of safety information in relation to dating apps because the information is not usually prominent and is often situated on separate websites and blog posts. This increases vulnerability to harm, coercion, and violence perpetrated online (Malta & Robards, 2017). Older women often lack knowledge about their internet security needs, making them more susceptible to deception, misrepresentation, and various scams, including romance scams (Cross, 2016). In 2018, the Office of eSafety Commissioner's survey of people over 50 years old found that over a quarter of the 3,602 participants (26%) had low digital literacy levels. Two-thirds of the women surveyed said they used their smartphone at least once a day. Notably, 45% of women indicated that they wanted to improve their competence in adjusting the privacy settings on their devices (2018). Such technology challenges are likely to translate to the dating app environment for this cohort of women.

We argue that older Australian women are a neglected group in dating app research, and a focus is needed to understand their particular safety concerns and the types of educational messaging that may be effective in helping older women to gain confidence in navigating dating apps and minimize their risk of becoming victims of DAFVA. Using a critical, feminist theoretical lens, the current study explored the dating app safety concerns of older Australian women and examined modalities and methods of effective dating app safety messaging for this cohort.

Funding for this project was provided by the Queensland government's Department of Justice and with this an accompanying expectation of (though not limited to) a regional intended audience. Two members of the research team worked in a small regional city, with connections to the North Queensland Women's Legal Service (NQWLS) and the local filmmaking community. These factors influenced the decision to adopt screen-based resources as the platform of choice for the project outcomes. Using a cocreation methodology and collaborative scripting model, this approach to resource development facilitated the creation of authentic diverse characters and narratives that targeted common scenarios encountered by women.

Research examining the suitability of short narratives for impact can be found in the field of entertainment-education (E-E) and specifically around narratives and behavior change. Factors such as personal interest, realism, and identification with main characters are important factors in narrative persuasion (Shin & Pettigrew, 2022; Van Leeuwen et al., 2016). In addition, cocreation is a suitable approach to content creation for specific cohorts, ensuring relatability and authenticity for the audience (Redvall, 2024). These approaches allowed the creation of identifiable characters, whose actions and responses were informed by lived experience and expert knowledge. Legal professionals from NQWLS who were actively engaged in cases dealing with technology-facilitated violence were a key cocreation partner.

## Methodology

The original study from which the data used in the current article were taken involved a mixed methods evaluation of how women aged 18 years old and above perceived a series of videos designed to educate viewers on how to navigate dating app usage safely. The project was approved by CQUniversity's Human Research Ethics Committee and was funded by Queensland Government's Office for Women and Department of Justice and Attorney-General, in partnership with the NQWLS. Internal funding was provided by CQUniversity's School of Business and Law along with in-kind support from the School of Education and the Arts.

One hundred and twenty-one women participants residing in Australia at the time of the study were recruited by Qualtrics using paid partner panels. Using partner panels limited the diversity of the sample but ensured validity of the data by avoiding false responses supplied by bots, which is becoming a pressing issue in online survey research (Horan et al., 2023). Participants were required to watch three character-driven, fictional videos filmed on location in Cairns, north Queensland, with each video focusing on a different aspect of dating app risk and safety. The videos can be viewed at: https://www.youtube.com/@DatingAppsSafety. The content of the videos was informed by deidentified case data supplied by the NQWLS to reflect currency and real-world issues encountered by women using dating apps.

Using a collaborative scripting approach, three main areas of concern and three different demographics were identified. The scripts were reviewed and refined by a group consisting of academics from the fields of arts, criminology, and law, lawyers from NQWLS, and actors were cast to reflect the demographics of the characters. The value of this approach to story and character development rests with its capacity to create authentic content and characters by integrating expertise and lived experience into scripts and the capacity to provide multiple opportunities for script revisions (Cattoni et al., 2022). The videos told the following stories:

### Video 1 Red Flags

A young woman coaches her friend who is reentering the dating scene on how to recognize “red flags” (potential warning signs) in online dating chats.

### Video 2 Show Us Your Bits

A professional woman is pressured to share intimate images of herself by a prospective date she is yet to meet. The message in this video reflects changes to the law in Australia regarding the sharing of intimate images without consent.

### Video 3 The Do's and Don’ts of Dating Apps

An older woman reentering the dating scene becomes the student when her daughter scrutinizes her online dating app practices.

For the current study, the sample included 56 participants aged 55–64 years old, 51 participants aged 65–70 years old, and 14 participants aged 75 years and above. There were limitations to the sample in terms of diversity. Although we attempted to recruit a mix of cisgender and transgender women, all survey respondents in these age categories were cisgender. We weighted the sample to ensure participants who were in the 55 to 75 years and above age categories were adequately represented in the research. Given the limited demographic data collected, we are unable to comment on the race and ethnicity, disability status, or socioeconomic status of participants. Participants were recruited from every Australian state and territory except the Australian Capital Territory and Tasmania.

Data were collected via the survey platform Qualtrics where participants were required to watch the three videos and then answer a series of questions based on their perceptions of the videos and their opinions about dating apps and safety more broadly. Participants could not access the survey questions until they had watched all three videos.

The survey contained both closed and open, free-text response questions in order to capture a diversity of perspectives about safety and dating apps. The closed questions were designed to collect quantitative data and focused on demographic information along with Likert scale questions focusing on participants’ perceptions of dating apps, dating app usage, helpfulness of the information, and whether watching the videos would influence their future behavior when using dating apps. The open-ended questions collected qualitative data on participants’ preferred video and storylines, relatability of the characters, most and least useful aspects of the videos, and the kinds of topics participants would like to see addressed in future videos. There was also space provided for participants to include any further comments about dating apps. The survey was designed to ensure participants received questions relevant to previous responses. The survey was open for a period of 3 weeks in November 2023 and participants were provided payment for survey completion by Qualtrics’ partner panels. The qualitative data for the current study were analyzed by the first author using reflexive thematic analysis (Braun & Clarke, 2019) from a feminist criminological positionality. Quantitative data were analyzed statistically and will be reported separately. The next section will present the qualitative findings arising from the data.

## Results

This section presents the salient themes for the cohort of older women as they relate to dating apps and risk and safety. Four themes will be discussed and supporting quotes will be used to illustrate modalities and methods of effective dating app safety messaging for these women. We will begin with themes related to dating app safety before moving on to themes related to the modality of presentation of safety information. The final theme presented in this article relates to behavioral change intention as a result of viewing the videos.

### Theme 1: Safety Concerns About Dating Apps

Older women consistently express heightened safety concerns with online dating. The potential dangers associated with dating apps, such as scams, inappropriate behavior, and violence, are significant considerations for these women. This demographic group emphasized the need for caution and vigilance when engaging with online platforms, and wanted clearer advice and information specifically tailored to their age group. “I have never used a dating app because of all the bad reports they get and the abuse of women. I would like to feel safe before I start to use one” (55–64 years old).

The data suggests that women in this age cohort actively seek information to stay aware of the potential risks associated with dating apps. External sources, such as media reports, play a role in shaping their understanding of online dating safety. This indicates a proactive approach to staying informed and educating themselves about the challenges posed by dating via apps. However, several participants acknowledged that media portrayals can be sensationalized and create an overly negative perception of dating app experiences. For example: “… more and more often you hear on the media about some poor woman being scammed of her savings or even stalkers out there preying on young women” (55–64 years old).

As a result of negative perceptions about dating apps, many participants explained that they preferred more traditional methods of meeting potential partners, such as through friends, family, or shared interests and hobbies. The value placed on in-person interactions and skepticism about the authenticity of online connections made via dating apps suggest this cohort prefers to rely on more familiar and conventional approaches to relationships and perceives these to be safer. These negative perceptions contribute to a reluctance to explore online dating, which, when coupled with fears about safety, led many participants to reject app usage outright. Some participants expressed disinterest, stating that they have never used dating apps and do not foresee doing so in the future. “I have never used a dating app. I have had no need to use one, but I know of people who have, and I don’t like them using the apps. I get scared for their safety” (65–74 years old).

While safety concerns and negative perceptions are common, some women in this age group shared success stories and positive experiences they experienced while using dating apps. These instances highlight that, despite reservations, meaningful connections and relationships can be formed through online platforms for women in this cohort. “I met my husband on a dating website. We have been together for ten years” (65–74 years old).

### Theme 2: Modalities of Safety Messaging Delivery

The three videos were designed to be a preventative measure and adopted a storytelling and humor-driven approach to educate viewers on how to navigate dating apps safely. In response to questions focused on the safety and risk-minimization messaging in the videos, respondents frequently mentioned that they preferred the video containing the mother and daughter duo, explaining that it was more informative for them and addressed relevant issues for their age group. While this video emerged as a common preference, there was still a considerable diversity in responses. Some participants had specific preferences for the other videos based on factors such as character relatability, humor, or the specificity of information provided. The varied responses regarding humor, video length, and storylines suggest that preferences among the audience in this cohort are diverse.

… the last one (mother and daughter) was best as it provided a lot of useful hints that a lot of people embarking on online dating wouldn’t know, especially people later in life. (55–64 years old) & I liked the teacher and the pussy because it was funny. But the last one was best as it provided a lot of useful hints that a lot of people embarking on online dating wouldn’t know, especially people later in life. (65–74 years old)

The recognition of the relevance of the content across different age groups is a recurring aspect of the responses. Participants appreciated videos that addressed concerns and scenarios applicable to various stages of life and particularly liked the use of the mother–daughter storyline. The positive dynamics between characters, such as friends or family providing support, added an empathetic and relatable dimension to the videos and were well-received. Participants appreciated the diversity of ages represented, including characters that reflected their own ages, allowing viewers to connect with the content, thus making the content more relatable, memorable, and reflective of real-life experiences. “The last one (mother and daughter) because I am a senior and probably wouldn’t have thought of any of these strategies” (65–74 years old).

The effectiveness of the storylines was noted across different age groups, with some participants specifically mentioning that they were relevant for both older and/or younger women. The realism and practicality of the characters resonated with viewers, describing them as regular people doing regular things in their everyday lives. Participants appreciated that the scenarios in the videos felt authentic, realistic, and believable for the women in this study.

They were normal people looking for normal relationships or companions (65–74 years old).

They (the videos) get the messages out in a humorous way that makes them more relatable and memorable. (65–74 years old)

Participants noted that the use of humor in the storylines across the three videos made the information more relatable and memorable, contributing to their effectiveness in delivering safety messaging. They expressed that humor and simplicity of delivery was key in conveying information, making the videos more engaging. However, while humor was generally appreciated by the majority of participants in the study, a few reported that the humor was crude or distasteful and was off-putting in terms of effective delivery of the safety information in the videos. A few participants expressed discomfort with certain graphic content, such as explicit (but pixelated) photographs in the storylines, suggesting that this type of content may impact the viewer's experience. “The 2^nd^ video is not useful at all, it's illicit” (65–74 years old).

### Theme 3: Using Safety Videos for Knowledge Building

The participants acknowledged that watching the videos has increased their knowledge and awareness of the potential risks and dangers associated with dating apps. Some participants reported that this newfound knowledge would influence their considerations for future use of dating apps to meet potential partners as they felt better equipped to manage interactions and were aware of what to be cautious of when interacting on dating apps. Participants found the storylines practically useful and appropriate for their age cohort, especially for those who may be inexperienced at online dating and were setting up dating profiles or responding to messages on dating apps.

The older woman was relatable to me, and if I were to try online dating, I’d probably be just as naive as her. (65–74 years old)

(I like) the last one, because the mother is closer to my age and needs help understanding what the risks are. (65–74 years old)

Many respondents appreciated the practical advice given in the videos, specifically tips on protecting themselves online, important considerations when creating dating profiles, and how to navigate moving from online to face-to-face interactions. Participants acknowledged the logical and useful advice provided and expressed feelings that their age groups are not usually supplied with quality and appropriate information about dating. “The last one … has good information without being patronising or full of unintelligible computer speak” (55–64 years old).

Many reported that the examples of “red flags” provided in one video, which are clues that indicate dishonesty and suspicious behavior or activities, were particularly useful. “All the tips about what to do and not do. Recognising clues in a profile that might indicate they are not being genuine” (55–64 years old). Participants also highlighted the signposting to a range of available resources and support services contained at the end of each video as helpful. “(I know) where to get help from if needed” (75 years old or above).

A significant number of participants mentioned that each video was informative and enabled them to reflect on different facets of safety when using dating apps. This suggests that the educational aspect of the content overall was well-received, regardless of specific preferences. The majority of participants in this cohort expressed positive comments about the educational value of the videos, specifically top tips, relatable examples, and the use of real-life scenarios to illustrate interpersonal dynamics. This particular cohort particularly appreciated the inclusion of specific details of technical advice, such as turning off location tracking while using the app. “They guide (on how) to use a dating app properly, the advice of dos and don’ts of dating, how to be safe when using a dating app” (65–74 years old).

The stories, characters and scenarios presented in the videos resonated with participants who related them to their own experiences and potential problems of using dating apps. This suggests that the relatability of the content enhances its effectiveness in conveying key safety messages. This includes the normalization of help-seeking and making sure to fully understand the safety features of the various dating apps. Some participants in the cohort advised that some advice provided was common sense and information that participants already knew was present in the videos. However, this also led to the recognition that such videos are necessary as many people in the 55+ demographic may lack this knowledge and frames of reference, and that learning from others’ experiences via the video storylines was helpful. “(I liked) all the tips about what to do and not do. Recognising clues in a profile that might indicate they are not good at being genuine. It was using women of different age groups” (55–64 years old).

### Theme 4: Behavioral Changes Among Older Women

Participants expressed a proactive approach to safety by outlining specific precautions they would now take on dating apps after viewing the set of safety videos. This includes avoiding sharing personal information, checking the safety features of dating apps, and being cautious about potential red flags in potential partners. Privacy is a recurring aspect of the data, with participants expressing intentions they would *now* take as a result of the content in the videos that they would not have taken previously. Many participants expressed that they will now exercise enhanced caution about the information they share on dating apps and will design their dating app profiles with privacy and safety in mind. This reflects a heightened awareness of the importance of safeguarding personal information and maintaining privacy after viewing the videos. “It (the videos) gave a list of things that you shouldn’t give out freely such as your address, where your workplace is and what your real name is” (55–64 years old).

Participants who expressed a willingness to use dating apps in the future often specified that they would now specifically check for safety features on the app they choose to use. This indicates a new, discerning approach, with individuals considering the security measures offered by different platforms and basing their choice of app on the safety features offered. This new, practical approach outlined by the women in the study aligns with a desire for tangible actions from internet safety organizations, dating app platforms, and the government to increase their personal safety while using dating apps. “If I go on a dating site again, I will definitely change some of the things I do” (65–74 years old).

Participants reported that the storylines made them think more about the potential dangers of using dating apps. This impact on viewer thinking affirms the educational value and awareness-raising benefits of the videos. The data demonstrate that the informational videos have had a concrete impact on participants’ behavior and/or behavioral intentions. Whether relating to adopting new safety measures when dating online, being more conscious of personal safety, or considering red flags in potential partners, the videos have influenced these women's approaches to using dating apps. Participants reported that they will be more vigilant after learning about the potential risks relevant to their age cohort when using dating apps directly as a result of watching the videos. “I would be looking out for red flags and not rushing into meeting anyone” (65–74 years old).

An unexpected outcome was that a small collection of women made the decision not to use dating apps at all. Several participants explicitly stated that they will not use dating apps, citing reasons such as lack of trust in the apps themselves, or their own negative previous experiences with online dating. There was a minority of women who expressed that the videos themselves had made them reluctant or unwilling to use dating apps in future, citing the range of possible risks and dangers presented in the videos as reasons behind this decision. “Haven’t used them and this survey has ensured I will never use them. I’m glad I’m not younger because this seems to be the main way to meet someone these days. Always women feeling unsafe, rarely the men” (55–64 years of age).

## Discussion

The findings of this study illuminate the nuanced relationship women aged 55 and over have with dating apps and highlight the diversity of attitudes toward such apps, and are discussed below.

### Lack of Safety and Legal Information

Our research highlighted a pervasive lack of awareness and access to information regarding safety measures, legal protections, and support services tailored to the unique needs of this cohort of women navigating digital dating environments. Women aged 55 and over, a demographic increasingly turning to dating apps (Ellin, 2014), face distinct vulnerabilities in these online spaces, exacerbated by societal attitudes, ageism, and digital literacy disparities (Jane, 2016). The findings of this study highlight a gap in the guidance available to women aged over 55 who engage with dating apps. Research has demonstrated that gender is an important factor in perceptions of risk of crime and mirror gender ideology (Choi & Merlo, 2021; Connell & Messerschmidt, 2005; Davis & Dossetor, 2010), and given that online attitudes and behaviors are extensions of offline social processes (Gillet, 2021; Jane, 2016; Licoppe, 2019; Lindsay, 2015), it is unsurprising that the women in our study are wary of dating apps, considering what steps they need to undertake to navigate dating apps safely (Berkowitz et al., 2021; Farvid & Aisher, 2016; Gillet, 2019) and thus are seeking additional information.

The findings of our study indicate that these women have significant concerns related to their personal safety while using dating apps, which reflect the prevalence in the media of the message that women's safety is at risk when using dating apps (Albury et al., 2020) and the “safety work” women undertake both online and offline (Berkowitz et al., 2021; Farvid & Aisher, 2016; Gillet, 2023, 2021; Vares, 2024).

Our research also indicates that older women are likely to seek out information on steps they can take to minimize risk. However, the women in the study reported that it was difficult to find educational resources that are relevant to them, and, despite being aware of some potential risks of dating apps such as romance or financial scams, practical information for this cohort was not readily available. In addition, there is limited information relating to the legal status of some online dating activities and conduct. Parliaments throughout Australia are aiming to respond to identified issues regarding both offline and online dating issues, and over the past 5 years, they have introduced numerous changes to criminal law in attempt to tackle them. For example, since 2019, it has been an offense to share intimate images without consent in Queensland. In 2024, the Queensland Parliament passed the *Criminal Law (Coercive Control and Affirmative Consent) and Other Legislation Amendment Act 2024*, which amended the Queensland Criminal Code to make “stealthing” an offense, which is the removal of or tampering with a condom during sexual activity when a party's participation was predicated on the use of a condom.

In response to calls for Queensland to move to an affirmative consent model, this legislation also amended the definition of consent to sexual activity and the operation of mistake of fact, which is where a defendant claims to be mistaken as to the victim's consent to sexual activity as a defense to rape or sexual assault. A further example of the rapid change experienced in this area is the passing of the *Criminal Code Amendment (Deepfake Sexual Material) Bill 2024* in August 2024, which amends the *Commonwealth Criminal Code* to criminalize the creation and distribution of AI-generated sexually explicit deep fake videos. However, there is a lack of available accessible information for older women that details these changes. Respondents’ commentary around the informative viewer-friendly nature of the resources provided in this project suggests that humor-driven videos focused on providing information through storytelling may provide an accessible, relatable medium for updating this cohort's knowledge about changes in the law. Such resources could be combined with guidance on how to seek assistance if one has been a victim of harassment or violence as a result of using dating apps.

### Modalities and Methods for Effective Educational Messaging

Many of the resources about dating apps and other forms of technology facilitated abuse currently available rely on a textual delivery (e.g., see https://www.esafety.gov.au/key-topics/staying-safe/online-dating). The short narrative films produced were low resource and able to be easily distributed online via the NQWLS website and via a dedicated YouTube channel. Compared to other forms of messaging, collaborative filmmaking is useful in highlighting complex issues and responses to situations that might be otherwise difficult to convey (Baumann et al., 2020). A fictitious online dating app was created and filmed for the resources that mirrored existing platforms. Onscreen characters were able to interact in real time with the app, with important information being conveyed with the use of graphics. The short films were able to recreate a credible experience for viewers in a memorable format. Within learning design, significant research has been undertaken to show that multimedia formats, including images and graphics, are more effective than text alone in information retention. Similarly, graphics can work to efficiently organize information (Clark & Mayer, 2024). Furthermore, harnessing information for social impact to a cohesive story can be significantly more impactful (Bublitz et al., 2016).

The videos were well received by most women in the cohort and were considered an effective method to deliver messaging about safety and risk because of the relevance of the information provided. In contrast, the Office of the eSafety Commission's survey reported that 78% of women reported that their preferred training method on how to use their digital devices was in-person classes or training from family or friends with only 28% very likely or extremely likely to use the eSafety Commissioner's online training portal (2018). It therefore appears that including relatable characters, age-appropriate information, realistic storylines, and humor in the videos may bridge the gap in educating older women who are new to or unfamiliar with dating apps. Further, the modality and content appeared to enhance the effectiveness of safety interventions and made the content relevant and memorable for this group of women.

The findings demonstrate that including relatable and authentic characters in educational materials can enhance engagement and credibility among this cohort of women about dating app risks and safety. Incorporating representations of women aged 55+ who reflect diverse backgrounds and experiences can help build rapport and foster a sense of connection with the target audience and increase the relevance of the storyline and the associated safety information. Audiences identify with characters they like, and this is useful because it means the audience enters the story through the character with whom they identify (Dancyger et al., 2023). For example, using narratives featuring other women as knowledgeable mentors or supportive peers can convey empathy and understanding while promoting positive modeling for safer online behavior. The videos also reflect older women's preference for learning about digital technologies through family members, friends and colleagues (eSafety Commissioner, 2018). Tailoring safety information to the unique needs and preferences of different age groups is crucial for effective communication. The participants in our study reported a lack of accessible, knowledge-appropriate information about dating app safety features and potential risks their age group specifically may encounter on dating apps such as financial scams. Recognizing that this cohort of women may have varying levels of digital literacy and familiarity with dating apps (Jorna, 2016), educational materials should be easy to locate, easy to understand, and relevant to their experiences. Providing visual rather than text-based safety information, including step-by-step guides, practical tips, and real-life examples tailored to women in this age group can empower them to navigate dating apps confidently.

### Promoting Behavior Change

Many of the women in this study reported that in the future they would alter their behavior when using dating apps or be mindful of the safety and risk issues identified in the videos as a result of watching the video resources. These findings demonstrate that engaging modalities of delivering safety information may positively influence future behavior by educating those who are looking for further information about risks and safety precautions, while enhancing self-efficacy and fostering empowerment through knowledge building. By providing clear and actionable guidance and safety interventions like the videos discussed in this article, we can equip women in this cohort with the knowledge to recognize warning signs, set boundaries, and seek support when encountering risks or threats on dating apps.

While it should not be incumbent on women to adopt safety strategies to reduce the likelihood of harm when engaging with dating apps, such practices have become normative for seasoned users of dating apps, just as women's “safety work” in traditional dating contexts is expected but unacknowledged (Gillet, 2023). It can of course be argued that in developing resources designed to educate women on these topics that we are in fact reinforcing the cultural expectation that women are responsible for ensuring that they are not victims of violence, rather than focusing on cultural change and accountability of the perpetrator (Vares, 2024). Logically, the question should then arise as to whether we really want to encourage any form of behavior change in women who are new to or unfamiliar with dating apps. However, the frequency of reports in the media of the broad range of harms correlated to dating app use encourages women to seek out information on how to minimize risk (Albury et al., 2020). If these women are going to seek such resources regardless of whether it is acceptable that society expects women to protect themselves from gendered violence, it is important that they feel empowered by the resources. This is particularly important when many women over the age of 55 have lower digital literacy levels and a lack of confidence in their technical knowledge and skills (eSafety Commissioner, 2018). Our research indicates that the modalities used in this research are more effective in providing older women with the information they are looking for. It should also be noted that this cohort lacks knowledge about security and privacy settings on apps more generally. Given that 45% of participants in the e-Safety Commissioner's survey identified they needed to learn more about this area (eSafety Commissioner, 2018), it can be argued that behavior change of this nature is inherently valuable as it brings older women to a similar level of competence in navigating security and privacy settings generally as their younger counterparts.

Some of the participants reported feeling empowered to engage with dating apps as a result of the videos. However, it should be noted that negative behavioral change was also an outcome for some participants, who indicated that the videos had introduced them to safety concerns or had reinforced their existing fears. While this is perhaps to be expected, these were a minority of the participants, and such comments must also be balanced against the fact that older women are less likely to be aware of security and privacy settings, geolocation tracking, and other technological aspects of using dating apps and are likely to actively seek out such information. Further, it should also be weighed against the fact that many of the women reported that the videos provided the content in an accessible and memorable way because they delivered the information in a constructive, humorous, and relatable manner.

It is apparent from the findings of this study that safety videos can serve as effective educational tools for this cohort of women. By presenting real-life scenarios, demonstrating warning signs, and offering strategies for risk reduction, the videos equip women with the knowledge and skills to recognize potential risks and respond in a practical and effective manner. By creating scripts that integrate research, expert guidance, and lived experience into actions and dialog, viewers can observe first-hand how characters might recognize and respond to a range of situations. We suggest that informational videos can enhance this age cohort's self-efficacy by showcasing examples of successful safety practices and modeling safer online behavior, in line with social cognitive theory (SCT; Bandura, 2003). SCT emphasizes the role of observational learning, self-efficacy, and social reinforcement in shaping behavior (Bandura, 2003), and using videos is an effective method to deliver this (Zhou et al., 2020). Safety videos can incorporate elements of SCT by including relatable and relational role models who demonstrate effective safety practices while using dating apps, modeling assertive skills such as enforcing boundaries and the pace of interactions or relationships, and providing opportunities for women in this age group to observe and learn from same-age peer experiences. This peer-to-peer element of educational messaging is currently lacking in available materials, according to the participants in the current study, who report that many dating app safety resources are aimed at teens and young women. By strengthening self-efficacy and fostering social support, safety videos can encourage older women to practise fully informed, safer online behaviors.

Viewing the videos prompted the participants in this study to plan to translate their new knowledge and awareness into tangible action, encouraging them to implement safety strategies in their own online dating interactions. By providing clear guidance, safety videos empower older women to take proactive measures that they were previously unaware of to minimize risk, such as verifying identities, setting privacy settings, and reporting abusive behavior.

Research has demonstrated that there is a clear gendered aspect to online interactions (Lazard, 2020). Feminist theorizations of the patriarchy elucidate how entrenched gender norms and power dynamics perpetuate inequalities in the offline world (Hunnicut, 2009) which is reproduced in digital environments and contributes to the lack of safety for older women on dating apps. In patriarchal societies, older women are often subjected to ageist stereotypes and devaluation (Bouson, 2016; Westwood, 2023), which can undermine their sense of agency and autonomy in online dating interactions, particularly for women already socialized into a passive dating role. Through the presentation of believable, engaging, and relatable video content, it is possible to promote meaningful behavior change that is counter to online gendered expectations and stereotypes, giving women more autonomy and control while using dating apps and empowering them to make informed decisions about risk and safety online.

### Limitations

A limitation of this study is the lack of diversity in the sample, as discussed earlier in the article. All 121 participants were cisgender, leading to a lack of data relating to transgender women's perceptions of and experiences with dating apps. It is reasonable to suggest that trans people, but trans women in particular, are required to engage in extra personal safety work when dating online due to additional risks of transphobia and associated violence (Griffiths & Armstrong, 2024). It is also likely that members of LGBTQ community use and experience dating apps differently from heterosexual people and have unique safety needs. Further research is needed on the information needs of gender-diverse and LGBTQ dating app users, while not conflating gender diversity with sexuality. In addition, further research on the experiences of marginalized groups, such as those with disabilities or from culturally and linguistically diverse backgrounds, requires further exploration. Other demographic data were not collected for the study to avoid additional participant burden, however, more details regarding specific participant ages would have been useful particularly for the 75 years and over cohort.

Given the participants were recruited via paid panels, there is the potential for self-selection bias. Participants who volunteer to participate in these panels may differ systematically from those who do not, leading to a lack of representation and participants may have financial motivations for participation which risks sampling bias.

The study focused on short-term outcomes immediately following exposure to the safety videos, without assessing long-term behavior changes or the sustainability of the intervention effects over time. Without follow-up assessments, it is challenging to determine whether the observed changes in knowledge, perceptions, or behaviors persist beyond the short term.

### Implications and Further Research

The implications of this study are multifaceted and apply to various stakeholders across community, organizations, and industry. The study findings inform the development of targeted interventions aimed at promoting online safety among this underserved cohort of women. The findings identify engagement strategies and content for effective safety messaging using videos, meaning researchers and practitioners can design evidence-based interventions and support resources tailored to the specific needs and preferences of older women, ultimately enhancing their ability to navigate digital dating environments confidently and from a more informed perspective.

The study findings can inform policy and practice initiatives in Australia aimed at addressing online safety issues among older adults. Policymakers, dating app developers, and community organizations can utilize the evidence generated by the study to advocate for legislative reforms, support services, technological innovations, and educational initiatives that prioritize the safety information literacy of women aged 55 and over. More broadly, videos, as opposed to text-based resources, can serve as educational tools for raising public awareness about online safety issues among older women. Public awareness campaigns, media outreach initiatives, and community workshops can disseminate safety videos to broader audiences, promoting dialog, understanding, and action around the importance of online safety for older women who use dating apps.

In terms of further research, it would be useful to assess the videos against other safety teaching techniques, such as instant messaging style text delivery or interactive AI chatbots, gamified safety apps, and augmented reality apps. Comparing methods of delivery would determine which approach leads to better retention of knowledge along with the method of delivery that improves confidence in terms of applying safety strategies in real-life situations. Delivery methods with more interactivity may suit a cohort of women who have higher levels of digital literacy, while less interactive character-driven simulations via video delivery may be more appropriate for those cohorts who are not digital natives. By comparing various delivery methods, it would be possible to blend the best elements of each method for specific cohorts of women.

## Concluding Thoughts

While this research has provided insight into the effectiveness of educational video resources designed for older women who are new to, unfamiliar with, or are considering using, dating apps, it should not be seen as placing the emphasis on older women, and women more generally, to protect themselves from harm while using dating apps. In isolation, such steps cannot address DAFSVA, given the date rape culture prevalent in society and replicated in the dating app environment. Placing responsibility on the dating app companies and governments is essential in this process while also tackling the root causes of DAFSVA. The government's Online Safety Code for dating services, effective from April 2025, is the first step in this process of focusing on the responsibility on tech companies. However, in light of the prevalence of DAFSVA, the voluntary nature of the Code is likely to reduce the impact it could have in shifting attitudes away from expecting women to engage in safety work. Despite these small steps, the reality is that women are actively seeking educational resources focused on how to minimize risk and harm when utilizing dating apps. Such resources are essential for those with low levels of digital literacy or are new to, or unfamiliar with, dating apps.

This study illustrates the significance of using educational safety videos as a means to empower women aged 55 years and older who use, or are considering using, dating apps. This responds directly to the lack of information tailored to the needs of this particular cohort, which is likely to have lower levels of confidence and technological skills. Careful consideration was given to the visual and narrative elements of the videos to ensure accessibility, engagement, and relevance for older women. The videos employed a character-driven format with age-relevant content, and the inclusion of relatable characters, real-life scenarios, and engaging storylines that enhanced engagement and retention of key safety messages. By promoting knowledge building, information accessibility, and fostering women's agency and autonomy, these videos serve as valuable tools for educating this cohort on dating app risk and safety. The findings highlight the importance of evidence-based, tailored interventions that acknowledge the unique needs and experiences of older women, while also informing policy and practice initiatives aimed at promoting a more inclusive and equitable digital dating landscape.

## References

[bibr1-10778012251366224] Albury K. McCosker A. Pym T. Byron P. (2020). Dating apps as public health ‘problems’: Cautionary tales and vernacular pedagogies in news media. Health Sociology Review, 29(3), 232–248. 10.1080/14461242.2020.1777885 33411606

[bibr2-10778012251366224] Australian Bureau of Statistics. (2022). Regional population by age and sex. https://www.abs.gov.au/statistics/people/population/regional-population-age-and-sex/latest-release

[bibr3-10778012251366224] Australian Institute of Health and Welfare. (2023). Australia’s welfare 2023: In brief. AIHW. https://www.aihw.gov.au/reports/australias-welfare/australias-welfare-2023-in-brief/summary

[bibr4-10778012251366224] Bailey J. (2012). Changing the game. The Age. https://www.theage.com.au/lifestyle/changing-the-game-20120211-1sy90.html

[bibr5-10778012251366224] Bandura A. (2003). Entertainment-education and social change. Routledge.

[bibr6-10778012251366224] Baumann S. E. Lhaki P. Burke J. G. (2020). Collaborative filmmaking: A participatory, visual research method. Qualitative Health Research, 30(14), 2248–2264. 10.1177/1049732320941826 32734829

[bibr7-10778012251366224] Ben-Ze’ev A. (2004). Love online: Emotions on the Internet. Cambridge University Press.

[bibr8-10778012251366224] Berkowitz D. Tinkler J. Peck A. Coto L. (2021). Tinder: A game with gendered rules and consequences. Social Currents, 8(5), 491–509. 10.1177/23294965211019486

[bibr9-10778012251366224] Bouson J. (2016). Shame and the aging woman: Confronting and resisting ageism in contemporary women’s writings. Palgrave Macmillan.

[bibr80-10778012251366224] Braun V. Clarke V. (2019). Reflecting on reflexive thematic analysis. Qualitative Research in Sport, Exercise and Health, 11(4), 589–597. 10.1080/2159676X.2019.1628806

[bibr10-10778012251366224] Bublitz M. G. Escalas J. E. Peracchio L. A. Furchheim P. Grau S. L. Hamby A. Kay M. J. Mulder M. R. Scott A. (2016). Transformative stories: A framework for crafting stories for social impact organizations. Journal of Public Policy & Marketing, 35(2), 237–248. 10.1509/jppm.15.133

[bibr11-10778012251366224] Byron P. Albury K. (2018). ‘There are literally no rules when it comes to these things’: Ethical practice and the use of dating/hook-up apps. In Dobson A. Robards B. Carah N. (Eds.), Digital intimate publics and social media (pp. 213–229). Palgrave Macmillan.

[bibr12-10778012251366224] Byron P. Albury K. Pym T. (2021). Hooking up with friends: LGBTQ+ young people, dating apps, friendship and safety. Media, Culture & Society, 43(3), 497–514. 10.1177/0163443720972312

[bibr13-10778012251366224] Calasanti T. (2008). A feminist confronts ageism. Journal of Aging Studies, 22(2), 152–157. 10.1016/j.jaging.2007.12.009

[bibr14-10778012251366224] Cama E. (2021). Understanding experiences of sexual harms facilitated through dating and hook up apps among women and girls. In Bailey J. Flynn A. Henry N. (Eds.), The emerald international handbook of technology-facilitated violence and abuse (pp. 333–350). Emerald Publishing.

[bibr15-10778012251366224] Cattoni J. Ryan C. Batty C. McAllister M. Nash J. (2022). Collaborative story development across the creative arts and nursing: Reflections on a practice-based filmmaking research project. Media Practice and Education, 23(4), 329–346. 10.1080/25741136.2022.2122682

[bibr16-10778012251366224] Choi J. Merlo A. V. (2021). Gender identification and the fear of crime: Do masculinity and femininity matter in reporting fear of crime? Victims & Offenders, 16(1), 126–147. 10.1080/15564886.2020.1787282

[bibr17-10778012251366224] Clark R. C. Mayer R. E. (2024). E-learning and the science of instruction: Proven guidelines for consumers and designers of multimedia learning (5th ed.). Wiley.

[bibr18-10778012251366224] Connell R. W. Messerschmidt J. W. (2005). Hegemonic masculinity: Rethinking the concept. Gender & Society, 19(6), 829–859. 10.1177/0891243205278639

[bibr19-10778012251366224] Cross C. (2016). ‘They’re very lonely’: Understanding the fraud victimisation of seniors. International Journal for Crime, Justice and Social Democracy, 5(4), 60–75. 10.5204/ijcjsd.v5i4.268

[bibr20-10778012251366224] Dancyger K. Keyt J. Rush J. (2023). Alternative scriptwriting: Contemporary storytelling for the screen (6th ed.). Routledge.

[bibr21-10778012251366224] Davis B. Dossetor K. (2010). (Mis)perceptions of crime in Australia. Trends and Issues in Crime and Criminal Justice, 396, 1–6. 10.52922/ti286081

[bibr22-10778012251366224] Debnam K. J. Kumodzi T. (2021). Adolescent perceptions of an interactive mobile application to respond to teen dating violence. Journal of Interpersonal Violence, 36(13–14), 6821–6837. 10.1177/0886260518821455 30600761

[bibr23-10778012251366224] De Ridder S. (2022). The datafication of intimacy: Mobile dating apps, dependency, and everyday life. Television & New Media, 23(6), 593–609. 10.1177/15274764211052660

[bibr24-10778012251366224] Dickson F. C. Hughes P. C. Walker K. L. (2005). An exploratory investigation into dating among later-life women. Western Journal of Communication, 69(1), 67–82. 10.1080/10570310500034196

[bibr25-10778012251366224] Dwyer Z. Hookway N. Robards B. (2021). Navigating ‘thin’ dating markets: Mid-life repartnering in the era of dating apps and websites. Journal of Sociology, 57(3), 647–663. 10.1177/1440783320948958

[bibr26-10778012251366224] Ellin A. (2014). Matchmakers help those over 60 handle dating’s risks and rewards. *The New York Times*. https://www.nytimes.com/2014/03/29/your-money/matchmakers-can-help-those-over-60-navigate-datings-risks-and-rewards.html

[bibr27-10778012251366224] eSafety Commissioner. (2018). Understanding digital behaviour amongst adults aged 50 years and over. https://www.esafety.gov.au/sites/default/files/2019-08/Understanding-digital-behaviours-older-Australians-full-report-2018.pdf

[bibr28-10778012251366224] Farvid P. Aisher K. (2016). ‘It’s just a lot more casual’: Young heterosexual women’s experiences of using Tinder in New Zealand. Ada: A Journal of Gender, New Media & Technology, 10, 1–18.

[bibr29-10778012251366224] Francis J. Ball C. Kadylak T. Cotten S. R. (2019). Aging in the digital age: Conceptualizing technology adoption and digital inequalities. In Ageing and digital technology: Designing and evaluating emerging technologies for older adults (pp. 35–49). Springer Nature.

[bibr30-10778012251366224] Franklin A. Barbosa Neves B. Hookway N. Patulny R. Tranter B. Jaworski K. (2019). Towards an understanding of loneliness among Australian men: Gender cultures, embodied expression and the social bases of belonging. Journal of Sociology, 55(1), 124–143. 10.1177/1440783318777309

[bibr31-10778012251366224] Furlo N. Gleason J. Feun K. Zytko D. (2021). Rethinking dating apps as sexual consent apps: A new use case for AI-mediated communication. In Companion Publication of the 2021 Conference on Computer Supported Cooperative Work and Social Computing (pp. 53–56).

[bibr32-10778012251366224] Gewirtz-Meydan A. Volman-Pampanel D. Opuda E. Tarshish N. (2024). Dating apps: A new emerging platform for sexual harassment? A scoping review. Trauma, Violence, & Abuse, 25(1), 752–763. 10.1177/15248380231162969 37036157

[bibr33-10778012251366224] Gillet R. (2019). Everyday violence: Women’s experiences of intimate intrusions on Tinder. [Doctoral dissertation, Queensland University of Technology].

[bibr34-10778012251366224] Gillet R. (2021). ‘This is not a nice safe space’: Investigating women’s safety work on Tinder. Feminist Media Studies, 23(1), 1–17. 10.1080/14680777.2021.1948884

[bibr35-10778012251366224] Gillet R. (2023). “This is not a nice safe space”: Investigating women’s safety work on Tinder. Feminist Media Studies, 23(1), 199–215. 10.1080/14680777.2021.1948884

[bibr36-10778012251366224] Griffiths D. A. Armstrong H. L. (2024). “They were talking to an idea they had about me”: A qualitative analysis of transgender individuals’ experiences using dating apps. The Journal of Sex Research, 61(1), 119–132. 10.1080/00224499.2023.2176422 36799719

[bibr37-10778012251366224] Hammack P. L. Hughes S. D. Atwood J. M. Cohen E. M. Clark R. C. (2022). Gender and sexual identity in adolescence: A mixed-methods study of labeling in diverse community settings. Journal of Adolescent Research, 37(2), 167–220. 10.1177/07435584211000315

[bibr38-10778012251366224] Henry N. Flynn A. Powell A. (2020). Technology-facilitated domestic and sexual violence: A review. Violence Against Women, 26(15–16), 1828–1854. 10.1177/1077801219875821 32998673

[bibr39-10778012251366224] Henry N. Powell A. (2018). Technology-facilitated sexual violence: A literature review of empirical research. Trauma, Violence, & Abuse, 19(2), 195–208. 10.1177/1524838016650189 27311818

[bibr40-10778012251366224] Horan K. A. Shoss M. K. Simone M. DiStaso M. J. (2023). Preventing and mitigating the influence of bots in survey research. In The sage handbook of survey development and application (pp. 306–324). Sage.

[bibr41-10778012251366224] Hunnicut G. (2009). Varieties of patriarchy and violence against women. Violence Against Women, 15(5), 553–573. 10.1177/1077801208331246 19182049

[bibr42-10778012251366224] Jane E. A. (2016). Misogyny online: A short (and brutish) history. Sage.

[bibr43-10778012251366224] Jorna P. (2016). The relationship between age and consumer fraud victimisation (trends & issues in crime and criminal justice No. 519). Australian Institute of Criminology.

[bibr44-10778012251366224] Kelly L. (1987). The continuum of sexual violence. In Hanmer J. Maynard M. (Eds.), Women, violence and social control (pp. 40–60). Humanities Press International.

[bibr45-10778012251366224] Kinsella K. He W. (2009). An aging world: 2008. Census Bureau.

[bibr46-10778012251366224] Lauckner C. Truszczynski N. Lambert D. Kottamasu V. Meherally S. Schipani-McLaughlin A. M. Taylor E. Hansen N. (2019). “Catfishing,” cyberbullying, and coercion: An exploration of the risks associated with dating app use among rural sexual minority males. Journal of Gay & Lesbian Mental Health, 23(1), 1–18. 10.1080/19359705.2019.1545205

[bibr47-10778012251366224] Lazard L. (2020). Sexual harassment, psychology & feminism #MeToo, victim politics and predators is neoliberal times. Springer.

[bibr48-10778012251366224] Licoppe C. (2019). Liquidity and attachment in the mobile hookup culture: A comparative study of contrasted interactional patterns in the main uses of Grindr and Tinder. Journal of Cultural Economy, 13(1), 73–90. 10.1080/17530350.2019.1607530

[bibr49-10778012251366224] Lindsay M. (2015). Performative acts of gender in online dating: An autoethnography comparing sites. In Alev I. Johnson J. Tao F. (Eds.), Online courtship: Interactions across borders (pp. 242–261). Institute of Network Cultures.

[bibr50-10778012251366224] Malta S. (2013). Why go online? Older adults’ reasons for online dating [Paper presentation]. The Australian Sociological Association National Conference ‘Reflections, Intersections and Aspirations: 50 Years of Australian Sociology’. Victoria, Australia: Monash University.

[bibr51-10778012251366224] Malta S. Farquharson K. (2014). The initiation and progression of late-life romantic relationships. Journal of Sociology, 50(3), 237–251. 10.1177/1440783312442254

[bibr52-10778012251366224] Malta S. Robards S. (2017). Older people as cyber-sexual beings: Online and internet dating. In Barrett C. Hinchcliff S. (Eds.), Addressing the sexual rights of older people theory, policy and practice. Routledge. (p.p. 139-153).

[bibr53-10778012251366224] Marston H. R. (2019). Millennials and ICT: Findings from the technology 4 young adults (T4YA) project: An exploratory study. Societies, 9(4), Article 80. 10.3390/soc9040080

[bibr54-10778012251366224] Marston H. R. Ivan L. Fernández-Ardèvol M. Rosales Climent A. Gómez-León M. Blanche T. D. Earle S. Ko P. C. Colas S. Bilir B. Öztürk Çalıkoğlu H. Arslan H. Kanozia R. Kriebernegg U. Großschädl F. Rohner R. (2020). COVID-19: Technology, social connections, loneliness, and leisure activities: An international study protocol. Frontiers in Sociology, 5, Article 574811. 10.3389/fsoc.2020.574811 33869500 PMC8022752

[bibr55-10778012251366224] McWilliams S. Barrett A. E. (2014). Online dating in middle and later life: Gendered expectations and experiences. Journal of Family Issues, 35(3), 411–436. 10.1177/0192513X12468437

[bibr56-10778012251366224] Mignault L. Vaillancourt-Morel M. Ramos B. Brassard A. Daspe M. (2022). Is swiping right risky? Dating app use, sexual satisfaction, and risky sexual behavior among adolescents and young adults. Sexual and Relationship Therapy, 39(3), 819–842. 10.1080/14681994.2022.2078804

[bibr57-10778012251366224] National Crime Agency . (2016). Emerging new threat in online dating: Initial trends in internet dating-initiated serious sexual assaults. https://www.nationalcrimeagency.gov.uk/who-we-are/publications/607-nca-scas-online-dating-report-2016/file

[bibr58-10778012251366224] Portolan L. McAlister J. (2022). Jagged love: Narratives of romance on dating apps during COVID-19. Sexuality & Culture, 26(1), 354–372. 10.1007/s12119-021-09896-9 34305389 PMC8294277

[bibr59-10778012251366224] Powell A. Henry N. (2016). Technology-facilitated sexual violence victimization. Journal of Interpersonal Violence, 34(17), 3637–3665. 10.1177/0886260516672055 27697966

[bibr60-10778012251366224] Powell A. Henry N. (2017). Sexual violence in a digital age. Palgrave Macmillan.

[bibr61-10778012251366224] Powell A. Henry N. (2019). Technology-facilitated sexual violence victimization: Results from an online survey of Australian adults. Journal of Interpersonal Violence, 34(17), 3637–3665. 10.1177/0886260516672055 27697966

[bibr62-10778012251366224] Pym T. Byron P. Albury K. (2021). ‘I still want to know they’re not terrible people’: Negotiating ‘queer community’ on dating apps. International Journal of Cultural Studies, 24(3), 398–413. 10.1177/1367877920959332

[bibr63-10778012251366224] Redvall E. N. (2024). Writing and producing for older teens: Aiming for authenticity, relatability, and co-creative strategies. In Writing and producing for children and young audiences (pp. 1–25). Palgrave Macmillan.

[bibr64-10778012251366224] Relationships Australia . (2017). Online dating. https://www.relationships.org.au/document/november-2017-online-dating/

[bibr65-10778012251366224] Rosenfeld M. J. Thomas R. J. (2012). Searching for a mate: The rise of the internet as a social intermediary. American Sociological Review, 77(4), 523–547. 10.1177/0003122412448050

[bibr66-10778012251366224] Rowse J. Bolt C. Gaya S. (2020). Swipe right: The emergence of dating-app facilitated sexual assault. A descriptive retrospective audit of forensic examination caseload in an Australian metropolitan service. Forensic Science, Medicine, and Pathology, 16(1), 71–77. 10.1007/s12024-019-00201-7 32026384

[bibr67-10778012251366224] Sharabi L. L. (2023). The enduring effect of internet dating: Meeting online and the road to marriage. Communication Research, 51(3), 259–284. 10.1177/00936502221127498

[bibr68-10778012251366224] Shin Y. Pettigrew J. (2022). Testing narrative persuasion of a culturally grounded, school-based “Dale se REAL” entertainment-education intervention and peer communication on Nicaraguan adolescent substance use. Journal of Health Communication, 27(4), 222–231. 10.1080/10810730.2022.2090030 35722984

[bibr69-10778012251366224] Smith A. Anderson M. (2016). 5 facts about online dating. Pew Research Center.

[bibr70-10778012251366224] Stanko E. (1990). Everyday violence: How women and men experience sexual and physical danger. Harper Collins.

[bibr71-10778012251366224] Tan S. (2017). Pining for love, passing on apps: Why are some singles in Australia hesitant about dating apps? YouGov. https://au.yougov.com/consumer/articles/49197-pining-for-love-passing-on-apps-why-singles-in-australia-hesitant-about-dating-apps

[bibr72-10778012251366224] Turley E. L. Corbett-Jarvis N. George A. J. McEwan A. (2023). The use of ‘consent apps’ in sexual encounters and their socio-legal implications: Why we need to know more. Psychology of Women and Equalities Review, 6(2), 24–34. 10.53841/bpspowe.2023.6.2.24

[bibr73-10778012251366224] Vandeweerd C. Myers J. Coulter M. Yalcin A. Corvin J. (2016). Positives and negatives of online dating according to women 50+. Journal of Women & Aging, 28(3), 259–270. 10.1080/08952841.2015.1137435 27191792

[bibr74-10778012251366224] Van Leeuwen L. Van Den Putte B. Renes R. J. Leeuwis C. (2016). Do narrative engagement and recipients’ thoughts explain the impact of an entertainment-education narrative on discouraging binge drinking? Media Psychology, 20(2), 194–220. 10.1080/15213269.2016.1142379

[bibr75-10778012251366224] Vares T. (2024). Women’s safety work on dating apps and rape culture. Feminist Media Studies, 1–15. 10.1080/14680777.2024.2442998

[bibr76-10778012251366224] Wada M. Clarke L. H. Rozanova J. (2015). Constructions of sexuality in later life: Analyses of Canadian magazine and newspaper portrayals of online dating. Journal of Aging Studies, 32, 40–49. 10.1016/j.jaging.2014.12.002 25661855

[bibr77-10778012251366224] Westwood S. (2023). “It’s the not being seen that is most tiresome”: Older women, invisibility and social (in)justice. Journal of Women & Aging, 35(6), 557–572. 10.1080/08952841.2023.2197658 37097812

[bibr78-10778012251366224] Wolbers H. Boxall H. Long C. Gunnoo A. (2022). Sexual harassment, aggression and violence among mobile dating app and website users in Australia, Research Report 25. Australian Institute of Criminology. https://www.aic.gov.au/sites/default/files/2022-10/rr25_sexual_harassment_aggression_and_violence_victimisation.pdf

[bibr79-10778012251366224] Zhou Q. Lee C. S. Sin S. C. J. Lin S. Hu H. Fahmi Firdaus Bin Ismail M. (2020). Understanding the use of YouTube as a learning resource: A social cognitive perspective. Journal of Information Management, 72(3), 339–359. 10.1108/AJIM-10-2019-0290

